# Effect of Black Tea Polysaccharides on Alleviating Type 2 Diabetes Mellitus by Regulating PI3K/Akt/GLUT2 Pathway

**DOI:** 10.3390/foods13121908

**Published:** 2024-06-17

**Authors:** Zhenbiao Zhang, Xuming Deng, Ruohong Chen, Qiuhua Li, Lingli Sun, Junxi Cao, Zhaoxiang Lai, Xingfei Lai, Zaihua Wang, Shili Sun, Lingzhi Zhang

**Affiliations:** 1 Department of Tea Science, College of Horticulture, South China Agricultural University, Guangzhou 510641, China; zhangzhenbiao@gdaas.cn (Z.Z.); 15280766637@163.com (X.D.); 2 Tea Research Institute, Guangdong Academy of Agricultural Sciences, Guangdong Key Laboratory of Tea Resources Innovation & Utilization, Guangzhou 510640, China; chenruohong@gdaas.cn (R.C.); liqiuhua@gdaas.cn (Q.L.); lingli_318@126.com (L.S.); junxic@126.com (J.C.); laizhaoxiang@gdaas.cn (Z.L.); laixingfei@gdaas.cn (X.L.); 3 Environmental Horticulture Research Institute, Guangdong Academy of Agricultural Sciences, Guangdong Provincial Key Lab of Ornamental Plant Germplasm Innovation and Utilization, Guangzhou 510640, China; wangzaihua@163.com

**Keywords:** black tea, tea polysaccharides, chemical property, hypoglycemic effect, PI3K-Akt

## Abstract

The bioactivity of tea polysaccharides (TPs) has been widely reported, but studies to date have focused on green tea. Some human health investigations have implied that black tea may possess potential antidiabetic effects, but less is known about their potential role and related antidiabetic mechanism. The present study was, therefore, conducted to investigate the chemical properties and antidiabetic activity of TPs from black tea. Monosaccharide composition revealed that Alduronic acid (77.8 mol%) considerably predominated in the fraction. TP conformation analysis indicated that three components in TPs were all typical of high-branching structures. Oral administration of TPs could effectively alleviate fasting blood glucose in type 2 diabetes mellitus (T2D) mice, with the values 23.6 ± 1.42, 19.6 ± 2.25, and 16.4 ± 2.07 mmol/L in the 200, 400, and 800 mg/kg·BW groups, respectively. Among these TPs groups, the 800 mg/kg·BW groups significantly decreased by 37.88% when compared with the T2D+water group (*p* < 0.05). Further studies demonstrated that TP treatment upregulated the expression of p-Akt/p-PI3K (*p* < 0.001). Additionally, TP treatment significantly promoted glucose transporter protein 2 (GLUT2) translocation in the liver (*p* < 0.001). These findings suggest that TPs from black tea protect against T2D by activating PI3K/Akt/GLUT2 signaling and might serve as a novel therapeutic candidate for T2D.

## 1. Introduction

Tea is one of the most popular nonalcoholic beverages, next only to water, and is consumed by more than two-thirds of the global population [1]. According to the degree of fermentation, tea can be classified into green (non-fermented), white, yellow (slightly fermented), oolong (semi-fermented), black (fermented), and dark (post-fermented) varieties [2], of which black tea, in particular, is the tea with the largest output and consumption all over the world. In contrast to other teas, the process used for the fermentation of black tea is believed to be critical for the formation of its unique characteristic aromas and flavors. Black tea has not only received significant interest for its unique sweetness and aroma but has also attracted attention for its healthy functions, such as its anti-obesity effect [3], antibacterial effect [4], and hepatoprotective effect [5]. Recently, there has been an increase in the number of researchers studying the mechanisms responsible for the quality, functional ingredients and biological activities of black tea.

With the rising incidence of type 2 diabetes mellitus (T2D), the antidiabetic effect of tea polysaccharides (TPs), ones of bioactive compounds isolated from tea, has received more and more attention. Many reports have indicated that TPs could decrease the blood glucose levels of diabetic mice (or rats) via oral administration [6,7]. Although it is well-known that TPs are the major bioactive constituent of tea that can exhibit effectively hypoglycemic activity on T2D, numerous studies have mainly focused on the antidiabetic effects and related mechanism of green tea or dark tea. For instance, in a study comparing four dark tea aqueous extracts (DTAEs) containing TPs, Liubao brick tea (LBT) and Pu-erh tea (PET) exhibited stronger antidiabetic activity by reversing insulin resistance [8]. The TPs derived from green tea significantly delayed glucose absorption by prohibiting the expression of a sodium-dependent glucose transporter, indicating that dietary supplementation of TPs could alleviate diabetes mellitus [9]. Although TPs were reported to possess superior hypoglycemic activity, there have been very few studies pertaining to the TPs of black tea. However, some human health investigations have implied that drinking black tea might be an effective adjunct to diabetes. A randomized, double-blind, placebo-controlled crossover study revealed that black tea consumption could decrease postprandial blood glucose compared with a placebo after sucrose intake [10]. In another study of 63,000 people aged 45 to 74 in Singapore, those who drank at least one cup of black tea per day had a 14% lower risk of diabetes than non-tea drinkers [11]. These observations indicate that black tea may possess superior antidiabetic activity, with the main bioactive compound being TPs.

Therefore, the present study aimed to extract TPs from black tea and explore the characteristics of different compositions and structures via chromatography using the DEP-cellulose 52-column, Sephadex G-100, Sephadex G-75 column and GPC-RI-MALS techniques. Furthermore, the potential effects and underlying mechanism on T2D were evaluated using an in vivo murine model to determine the healthy function of TPs in black tea.

## 2. Materials and Methods

### 2.1. Materials

Yinghong No. 9 black tea was purchased from the Tea Research Institute, Guangdong Academy of Agricultural Sciences of China (Guangzhou, China). Streptozotocin was purchased from Sigma-Aldrich (St. Louis, MO, USA). The remaining chemicals and solvents for this study were of analytical grade.

### 2.2. Tea Polysaccharides Extraction and Purification

TPs were prepared using decolorization and hot water extraction. The dried Yinghong No. 9 black tea leaves were crushed into 40-mesh tea powders. These dried tea powders were then extracted (the ratio of liquid to solid 5:1, *v*/*v*) by ultrasonic vibration with 85% ethanol for 30 min in a room-temperature environment. The upper layer of ethanol was poured off, and the tea powders were repeatedly decolorized until the upper layer of ethanol was transparent. The decolorized tea powders were dried in a 50 °C oven and reserved.

The abovementioned decolorized tea powders were immersed in hot water at 60 °C for 90 min (solid–liquid ratio 1:20). After filtration, all extraction solutions were collected and concentrated to 1/5 volume under reduced pressure. Absolute ethanol was slowly added to the concentrate so that the final concentration was 80%, and then it was left to stand at 4 °C overnight. Then, the resultant precipitate was centrifuged (4000 rpm, 15 min); collected; washed alternately with acetone, diethyl ether, and absolute ethanol more than 3 times; and redissolved in distilled water. The solution was treated using the Sevage method to remove proteins (1/3 or 1/4 volume of Sevage reagent (chloroform–n-butanol volume ratio of 5:1) was applied), and then the mixture was vigorously shaken for 20 min. The mixture was centrifuged (4000 rpm, 15 min) to obtain the supernatant and then freeze-dried into crude TPs.

### 2.3. Determination of Monosaccharide Contents of TPs

Five milligram (±0.05 mg) samples of TPs were weighed accurately, the prepared trifluoroacetic acid solution was added, and the samples were heated for 2 h at 121 °C. This process was repeated two to three times, and samples were blown dry with nitrogen and cleaned with methanol. Finally, the obtained sample was dissolved in sterile water and transferred to a chromatography bottle for testing. The chromatographic conditions were as follows: an ion chromatographic instrument (ICS5000, Thermo Fisher Science, Waltham, MA, USA) was used; the column was DionexTM CarboPac (Thermo Fisher Science, USA); and the mobile phase was ddH_2_O in phase A, 100 mmol/L NaOH in phase B, and 100 mmol/L NaOH/200 mmol/L NaAc in phase C. The flow rate was 0.5 mL/min.

### 2.4. Analysis of TP Conformation Using the GPC-RI-MALS Technique

After dissolving the TPs in water, DEP-cellulose 52-column chromatography (2.6 cm × 70 cm) was used to elute it with NaAc-HAc buffer salt (pH 5.2) containing 0, 0.1, and 0.3 mol/L NaCl. Then, the three components were obtained from TPs using the phenol–sulfuric acid tracer method, subjected to repeated Sephadex G-100 (with dimensions of 2.6 cm by 90 cm) and Sephadex G-75 (with dimensions of 2.6 cm by 90 cm) column chromatography and then eluted in distilled water to obtain TP-A, TP-B, and TP-C.

In this experiment, the GPC-RI-MALS (high-temperature gel permeation chromatography 18 angle laser light scattering test) analysis method was adopted to detect the TP samples. The specific analysis conditions and analysis methods were as follows: the detectors were RI: Optilab T-rEX (Wyatt Technology, Menlo Park, CA, USA), MALS: DAWN HELEOS (Wyatt Technology, CA, USA), and PUMP: Series 1500 Pump, water; the mobile phase was 0.1 mol/L NaNO3, the flow rate was 0.4 mL/min, and the column temperature was 60 °C; the analytical column models were Ohpak SB-806 HQ, Ohpak SB-806 HQ, and Ohpak SB-804 HQ; and the sample load was 100 µL. TPs samples were accurately weighed and prepared into a 5 g/L solution using the mobile phase, and then the mixture was filtered using a 0.22 µm filter to analyze GPC-RI-MALS.

### 2.5. Animals

All experimental procedures were carried out in the low-temperature environment according to the institutional guidelines for the use of laboratory animals, with every effort made to reduce animal suffering. The Animal Care & Welfare Committee of Tea Research Institute, Guangdong Academy of Agricultural Sciences approved the protocol (ethical number: [2022] No. 069).

This study used 164 male C57BL/6J mice aged 4 weeks (from Beijing Vital River Laboratory Animal Technology Co., Ltd, Beijing, China). During the whole experimental period, all animals were allowed to freely obtain water and food. The mice were stored in a specific laboratory without pathogens, and the standard conditions were relative humidity (55 ± 5%), temperature (23 ± 2 °C), and light–dark cycle (12/12 h).

### 2.6. Induction of Type 2 Diabetes Mellitus

The study used high-fat feed with a low, single dose of streptozotocin to induce type 2 diabetes mellitus [12]. After acclimating for 7 days, the mice were randomly classified into 2 groups. One group of mice was fed a normal diet and kept as a normal control. The diabetic mice were fed a diet containing high fat (abbreviated as HFD; the percentage of total kcal was 16.46% protein, 37.89% carbohydrates, 45.65% fat) and induced ad libitum for 4 weeks. This diet was given until the experiment ended. After four weeks of dietary control, for the mouse group fed the high-fat diet, a single dose of streptozotocin (STZ) was injected [murine body-weight dosage was 80 mg/kg in 0.1 M pH 4.5 citrate buffer, intraperitoneally (i.p.)] without fasting. For the mice in the healthy control group, equivalent amounts of normal saline and citrate buffer were administered. Seventy-two hours after STZ injection, the model mice were fasted for 12 h, and their blood glucose concentrations were measured to ensure that diabetes mellitus (DM) was induced in all mice. The blood glucose concentration was 11.10~20.00 mmol/L, and the concentration was still stable after one week. These mice were considered to have diabetes and were selected for further subsequent experiments.

### 2.7. Experimental Design

An optimal dose evaluation experiment on TPs was conducted before the present experiment, and the results demonstrated that the therapeutic effect of TPs showed a trend of enhancement with the increase in concentration, reaching the best therapeutic effect at 800 mg/kg, and then a trend of decline after 800 mg/kg. Therefore, 800 mg/kg was used as the highest dose of the mouse administration concentration, and the doses of 200 mg/kg and 400 mg/kg were set downward based on the proportional relationship. Type 2 diabetes mellitus mice were randomized into five groups with 20 mice in each group. Diabetic control group: type 2 diabetes mellitus control mice were administered an equal amount of distilled water daily; positive control group: diabetic mice were administered metformin hydrochloride (250 mg/kg·BW, p.o. Three tea polysaccharide treatment groups were established according to the following methods: Diabetic mice were administered tea polysaccharides at 200, 400, and 800 mg/kg·BW (p.o.) per day, respectively. Healthy mice were considered a normal control group and administered an equal amount of distilled water. Treatment was continued for 60 days. During the whole process, the normal mice were given a normal diet, while the diabetic mice were given an HFD. The animals exposed to tea polysaccharides were not found to have side effects in behavior and general health. The body weight and total consumption of food and water were monitored every 3 days and varied between the groups. Serum insulin levels and fasting blood glucose were measured every 30 days.

### 2.8. Oral Glucose Tolerance Test

After 30 days of intervention, this assay was conducted on mice that had been fasted for 12 h through oral administration of glucose at a dose of 2.0 g/kg·BW. Blood was sampled from the tail vein to determine plasma glucose using a glucometer at specific time points of 0, 15, 30, 60, 120, and 240 min.

### 2.9. Blood Sampling and Preparation of Tissue Homogenate

All mice were fasted overnight upon the completion of the last administration. The mice were exposed to general carbon dioxide and, thus, sacrificed, and then blood samples were collected using the cardiac puncture technique. The serum was kept for 30 min under room temperature conditions, centrifuged for 20 min at 3500 rpm, separated, and then kept at −80 °C until further use. Normal saline was perfused transcardially to the animals through the ascending aorta to remove the blood clot from tissues and organs. The liver was removed quickly, weighed, and washed thoroughly using phosphate-buffered saline (PBS, pH 7.4). After quickly removing half of the large lobe of the liver, it was then kept in 10% buffered formalin solution for histopathological examinations; after being homogenized in ice-cold, phosphate-buffered saline, the remaining part was then stored in liquid nitrogen for different biochemical and molecular assays. The entire procedure was conducted at a low temperature.

### 2.10. Serum Biochemical Analysis

The levels of fasting blood glucose from the tail vein and glycemic load (GL) were measured using a OneTouch^®^ UltraEasy^®^ blood glucose meter from Johnson & Johnson (New Brunswick, NJ, USA). To better quantify variations in fasting blood glucose between the groups, the glucose tolerance test (GTT) and the area under the curve of the GTT (AUC of the GTT) were used. Commercial assay kits were used to measure total cholesterol (TC), triglyceride (TG) and low-density lipoprotein cholesterol (LDL-C), and high-density lipoprotein cholesterol (HDL-C). Operation was carried out in accordance with the kit instructions (total cholesterol assay kit, item No.: A111-1-1; triglyceride assay kit, item No.: A110-1-1; low-density lipoprotein cholesterol assay kit, item No.: A113-1-1; and high-density lipoprotein cholesterol assay kit, item No.: A112-1-1; Jiancheng Bioengineering Institute, Nanjing, China). Insulin resistance and sensitivity were evaluated by assessing the homeostasis model of insulin resistance (HOMA-IR) and sensitivity (HOMA-IS) and were calculated as follows: HOMA-IR was calculated using the formula fasting serum insulin (mU/mL) × fasting plasma glucose (mmol/L)/22.5; HOMA-IS was calculated using Formula 1/[fasting plasma glucose (mmol/L) × fasting serum insulin (mU/mL) [13].

### 2.11. ELISA for Serum Insulin

The amount of insulin in serum was measured using commercial ELISA kits, according to the manufacturer’s instructions.

### 2.12. Protein Extracted from Analysis of the Liver and Western Blot

The liver tissues (right lobe, 30 mg) from mice of each group were homogenized on ice for 10 s using a polytron tissue homogenizer; later, they were lysed in 0.5 mL ice-cold lysis buffer [50 mmol/L Tris (pH 7.4), 150 mmol/L NaCl, 1% Triton X-100, 1% sodium deoxycholate, 0.1% SDS, proteinase inhibitor (Roche Applied Science, Penzberg, Germany) and phosphatase inhibitor (Sigma-Aldrich, St. Louis, MO, USA)]. The mass ratio of tissues to lysis buffer was 1:99. Protein on the cell membrane was extracted using a Membrane Extraction Kit (Sigma-Aldrich, St. Louis, MO, USA). The samples obtained above were subjected to Western blot analysis, which followed the method reported in our previous study. Rabbit polyclonal antibodies (1:500) were used to detect the p-AKT, p-PI3K, and p-GSK-3β mammalian targets of rapamycin (mTOR), Rictor, and glucose transporter protein 2 (GLUT2) (Cell Signaling Technology, Danvers, MA, USA). The normalized results of the expression levels of those proteins were β-actin expression (internal control). Mouse polyclonal antibody, with a ratio of 1:2000, was used to detect β-actin (Sigma-Aldrich).

### 2.13. Immunofluorescence Analysis of p-Akt in the Liver

Ten percent formaldehyde was perfused intracardially into liver tissues for quantitative immunofluorescence (IF). Formalin was used to fix liver tissues, which were then embedded in paraffin and sliced. Slides were deparaffinized with xylene, quenched using hydrogen peroxide, and blocked in normal goat serum (Invitrogen, Life Technologies, Waltham, MA, USA) at a concentration of 10% at room temperature for 1 h. Then, the p-Akt antibody was used to incubate the sections overnight. After incubation with IF Detection Reagent (Cell Signaling Technology, Danvers, MA, USA) against rabbit IgG, the sections were incubated for 60 min and then developed with 4′,6-diamidino-2-phenylindole (DAPI) to obtain the nuclei. An Olympus BX60 microscope (Tokyo, Japan) with a color camera was used to take the images.

### 2.14. Statistical Analysis

All tests were conducted in triplicate, and the values are shown as the mean ± standard deviation. One-way ANOVA was applied to compare the means, and then GraphPad Prism 7.0 software for Windows was used for Tukey’s comparisons (GraphPad Software Inc., San Diego, CA, USA). The confidence level for significance was set at *p* < 0.05.

## 3. Results

### 3.1. Components of Hydrolyzed Monosaccharides and Molecular Configuration of TPs

The monosaccharide composition of TPs is shown in Table 1. Alduronic acid was the predominant component in TPs, accounting for 77.8%. Additionally, it can be seen that glucose was the highest neutral monosaccharides in TPs, accounting for 1.92%. As a result, the tea polysaccharide extracted from black tea was an acid polysaccharide.

The molecular weight with average weight (Mw: tensile strength), molecular weight with average number (Mn: kinetics studies and stoichiometric calculation) distribution, root mean square (RMS) radius of gyration (RMS-Rg), and polydispersity (Mw/Mn) of the three components (TP-A, TP-B, and TP-C) in TPs were measured. The conformation of the three components in the solution was calculated using the relationship between RMS-Rg and Mw. In Table 2, TP-A is the largest Mw (70.9 kDa) and has the widest range of relative molecular mass distribution (polydispersity Mw/Mn is 7.004 ± 23.444%); the Mw of TP-B is second (67.5 kDa), but the RMS-Rg of TP-B is largest (28.0 ± 19.8% nm); and TP-C has the smallest Mw (67.1 kDa) and polydispersity Mw/Mn (4.716 ± 3.159%). In the mobile phase, the conformation of polymers in different solvents is used for analysis according to the conformation plot. With the log (molar mass) as the abscissa and the log (R.M.S. radius) as the ordinate, the slope can reflect the molecular configuration of the polymer. Since the conformation plot slopes of three TPs components (TP-A, TP-B, and TP-C) are 0.20, 0.18, and 0.17 and the curve fitting of the three components are similarly U-shaped, these results indicate that they are typical of a high-branching structure.

### 3.2. Effect of TPs on Body Weight, Water, and Diet Consumption

**Figure 1:**
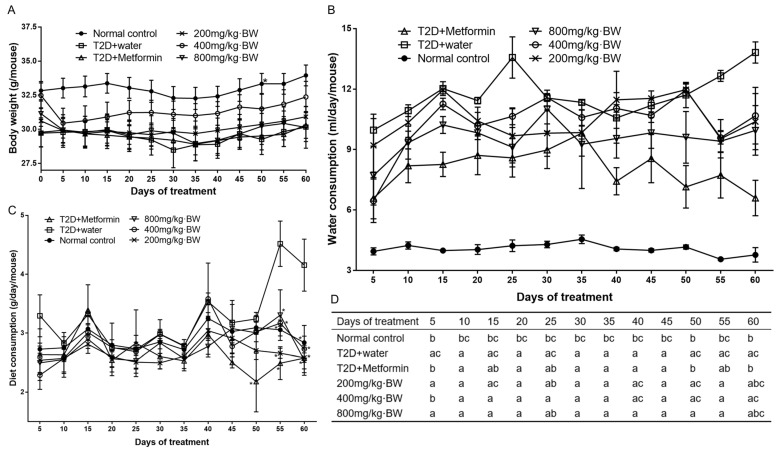
(**A**) Effect of TPs treatment on body weight of each group of mice. (**B**) Effect of TPs treatment on water consumption of each group of mice. (**C**) Effect of TPs treatment on diet consumption of each group of mice. (**D**) List of results of one-way ANOVA of values in the diet consumption among different groups. Data are expressed as means ± SD (n = 9). * *p* < 0.05 as compared to the T2D+water group.

Before starting treatment, there was no significant difference in body weight or consumption of water and diet among the different groups. Diabetic mice began to lose body weight after injecting STZ and feeding a high-fat diet for 35 days, and the consumption of water and diet began to increase sharply, which indicated that the T2D model was successfully established. After 55 days of the administration of TPs and metformin, the consumption of food and drinking water for the mice in the drug-administered group decreased significantly compared with the T2D+water group (Figure 1B–D). While the average body weight of the drug-administered group did not change significantly, implying that the T2D symptoms of mice were relieved (Figure 1A).

### 3.3. Effects of TPs on Glucose Tolerance Profiles

**Figure 2:**
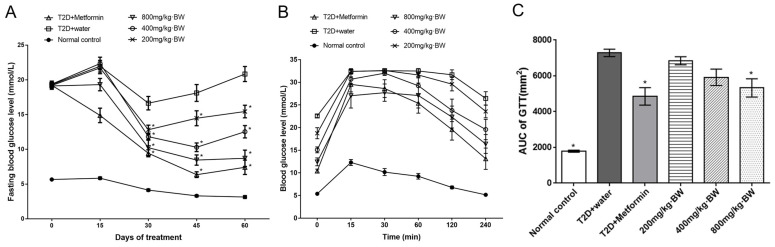
(**A**) Effect of TP treatment on blood glucose levels in each group of mice. (**B**) Effect of TP treatment on OGTT blood glucose levels in each group of mice. (**C**) AUC (Area under the curve) of the OGTT. Data are expressed as means ± SD (n = 9). * *p* < 0.05 as compared to the T2D+water group.

After successful induction of T2D, the indicated TPs administration was started. The fasting blood glucose of the treated diabetic mice began to decrease after a month and this trend continued until the end of the trial. The data showed that the FBG levels after 30 days of TPs and metformin intervention were 3.3 ± 0.14, 16.6 ± 0.97, 9.4 ± 0.50, 12.83 ± 0.65, 11.8 ± 0.52, and 10.2 ± 1.20 mmol/L in the normal control group, T2D+water, T2D+metformin, and 200, 400, and 800 mg/kg·BW groups, respectively (Figure 2). It is worth noting that FBG levels were significantly inhibited by TPs in a dose-dependent manner, of which the 800 mg/kg·BW group possessed the best treatment effect.

The tail vein blood at various time points, including 0, 15, 30, 60, 120, and 240 min, was sampled and measured using a blood glucose meter to characterize the status of glucose tolerance profiles. Glucose at a dose of 1 g/kg·BW was administered to mice given the indicated treatments by oral gavage after 12 h of fasting. At 15 time points, values for the T2D+water, T2D+metformin, and 200, 400, and 800 mg/kg·BW groups were significantly higher, by 1.63-, 1.40-, 1.63-, 1.60-, and 1.20-fold (*p* < 0.05), respectively, than the normal control group, and the 800 mg/kg·BW group decreased by 16.67% compared with the T2D+water group. The glucose assimilation peak in most diabetic mice was 30 min. At this time point, the values for the T2D+water, T2D+metformin, and 800 mg/kg·BW groups were significantly higher by 2.20-, 1.81-, and 1.72-fold, respectively, than the normal control group, and the 800 mg/kg·BW group significantly decreased by 14.87% compared with the T2D+water group. No significant difference existed among the T2D+water and 200 and 400 mg/kg·BW groups after 60 min of glucose administration. The T2D+water group still had a significantly higher glucose content than the normal control group by 2.53-fold. However, the T2D+metformin and 800 mg/kg·BW groups significantly decreased by 21.85% and 16.62%, respectively, compared with the T2D+warter group. At the 240 min time point, the blood glucose values were 5.2 ± 0.13, 26.4 ± 1.49, 13.1 ± 2.36, 23.6 ± 1.42, 19.6 ± 2.25, and 16.4 ± 2.07 mmol/L in the normal control, T2D+water, T2D+metformin, and 200, 400, and 800 mg/kg·BW groups, respectively. The blood glucose of T2D+water still maintained a significantly higher concentration than the normal control by 3.01-fold. In addition, the T2D+metformin and 800 mg/kg BW groups also significantly decreased by 50.38% and 37.88%, respectively, compared with the T2D+water group. The AUC of the indicated treatments was calculated based on individual glucose profiles (Figure 2C). The AUC index of the T2D+water group was significantly higher than that of the normal control by 3.07-fold, while the AUCs of the T2D+metformin and 800 mg/kg·BW groups were significantly decreased by 33.57% and 26.82%, respectively, compared with that of the T2D+water group. The above results demonstrated that administration with TPs could significantly decrease the fasting blood glucose of T2D mice in a dose-dependent manner. TPs may possess superior hypoglycemic activity.

### 3.4. Effects of TPs on Fasting Blood Glucose and Insulin Tolerance in Serum

**Figure 3:**
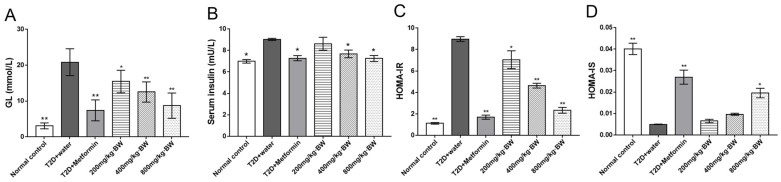
(**A**) Effect of TP treatment on fasting blood glucose levels in each group of mice. (**B**) Effect of TP treatment on serum insulin levels in each group of mice. (**C**) Effect of TP treatment on insulin resistance in each group of mice. (**D**) Effect of TP treatment on insulin sensitivity in each group of mice. Data are expressed as means ± SD (n = 9). * *p* < 0.05 and ** *p* < 0.001 as compared to the T2D+water group.

The effects of TPs in diabetic mice were then investigated, and it was determined that insulin sensitivity was increased and insulin resistance was reduced. The fasting blood glucose and insulin secretion were tested in the mice after 60 days of drug treatment, as shown in Figure 3A,B, further calculating the values of HOME-IR and HMOE-IS using the formula (Figure 3C,D). Compared with the T2D+water group, the lower HOMA-IR values were significant and negatively correlated with doses of TPs groups (*p* < 0.05; Figure 3C). Additionally, HOMA-IS values were significantly increased in the T2D+metformin and 800 mg/kg·BW (of TPs) groups relative to the T2D+water group. In these results, TPs showed the ability to improve insulin resistance in diabetic mice. These results suggest that TPs may exert hypoglycemic activity by improving insulin resistance in diabetic mice.

### 3.5. Effects of TPs on Lipid Levels in Serum

**Figure 4:**
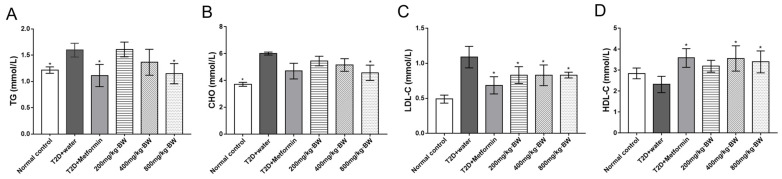
Antihyperlipidemia effects of TPs in each group of mice on serum lipid levels: (**A**) triglyceride; (**B**) total cholesterol; (**C**) low-density lipoprotein cholesterol; and (**D**) high-density lipoprotein cholesterol. Data are expressed as means ± SD (n = 9). * *p* < 0.05 as compared to the T2D+water group.

To further evaluate whether TPs can improve diabetic phenotypes, the lipid levels in mouse serum were investigated. The TPs effects on different serum biochemical parameters are presented in Figure 4. The TG conditions of the T2D+water groups were worse than those of the control group (*p* < 0.05). The results showed that TG levels significantly decreased by 38.68% and 27.98% in the T2D+metformin and 800 mg/kg·BW groups, respectively, in contrast with the T2D+water groups. The serum CHO levels were significantly decreased by 23.80% in 800 mg/kg·BW group mice compared with T2D+water group mice (Figure 4B). T2D mice, after TP and metformin administration, showed that LDL-cholesterol (*p* < 0.01) levels escalated significantly and HDL-cholesterol (*p* < 0.01) levels were depleted simultaneously. LDL was significantly decreased by 23.55%~37.00% in the mice of all drug administration groups in comparison with the T2D+water group, which was almost equal to that of the normal control group (Figure 4C). Interestingly, HDL was increased by 53.44% and 46.55% in mice in the 200 and 800 mg/kg·BW groups, respectively, compared with that in the T2D+water group (Figure 4D). These observations indicated that treatment with TPs could improve the lipid levels in mouse serum.

### 3.6. Effects of TPs on the Akt Signaling Pathway in the Livers of Diabetic Mice

**Figure 5:**
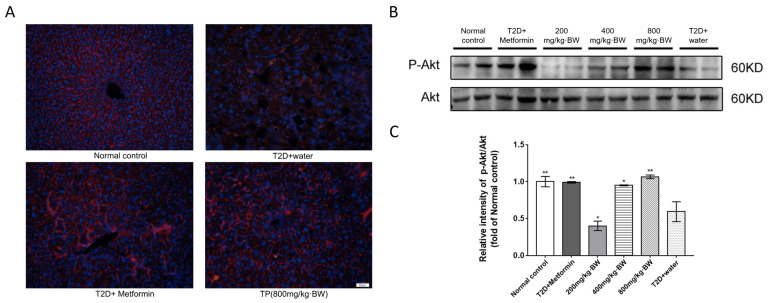
TP treatment increased the level of phosphorylated Akt in each group of diabetic mice: (**A**) immunofluorescent staining for p-Akt in liver sections; (**B**) Western blot analysis of p-Akt and Akt protein levels in mouse liver; and (**C**) relative expression of p-Akt to Akt levels. Data are expressed as means ± SD (n = 9). The scale bar represents 50 μm. * *p* < 0.05 and ** *p* < 0.001 as compared to the T2D+water group.

The pathogenesis of T2D is a process that activates complex molecular pathways. Existing evidence suggests that Akt/PKB plays a key role in hyperglycemia and reduces glucose transport in muscle tissues while inhibiting the release of glucose from hepatocytes [14]. Phosphorylated Akt, in an activated state, was able to promote muscle and adipose tissues to take up glucose stimulated by insulin while inhibiting the release of glucose from hepatocytes. Insulin affects the glucose uptake of peripheral tissues through Akt/PKB by translocating GLUT to the cell membrane, thereby promoting glucose uptake [15]. In this study, Western blot analysis showed that the protein expression of p-Akt in the liver of diabetic mice treated with TPs (0.95- and 1.06-fold of normal control mice in the 400 mg/kg·BW group and 800 mg/kg·BW group, n = 9) was increased significantly compared with that in the T2D+water group (0.59-fold of normal control mice, n = 9, *p* < 0.05). Strongly positive immunoreactivity for p-Akt was observed in the livers of TP-treated and metformin-treated diabetic mice, whereas biopsies from T2D+water mice were negative and different from normalcy (Figure 5A), indicating that TPs might exert hypoglycemic activity by inhibiting the phosphorylation of Akt.

When insulin binds to its cell surface protein, the receptor will subsequently phosphorylate its tyrosine, causing insulin receptor substrates to be phosphorylated on specific tyrosine residues and simultaneously activating PI3K (PI3 kinase), which is upstream of Akt. In skeletal muscle cells, AS160 of the Akt substrate (also called TBC1D4) is a qualified candidate in GLUT2 translocation, introduced through the Akt/PKB signaling pathway. The reason for GLUT2 being retained in GLUT storage vesicles was that AS160 was able to maintain Rab-Gpase in an inactive state via guanosine-50-diphosphate loading. AS160 is phosphorylated when Akt is activated, thereby reducing Rab-GAP activity and promoting GLUT2 transfer from vesicles, resulting in increased uptake of glucose. Therefore, any defect in the PI3K/Akt/AS160 transduction pathway was closely related to the uptake of glucose in skeletal muscle cells. Similarly, defects in Akt in liver cells can also cause glucose tolerance exceptions, insulin resistance, and so on. The possible involvement of PI3K in the effects of TPs on diabetic mice was further examined. After TP or metformin treatment of diabetic mice, significant escalation (*p* < 0.05) was observed in the levels of PI3K compared with the T2D+water group (1.31-, 0.89, 1.19-, 1.25-, and 0.53-fold of normal control mice in the T2D+metformin, 200 mg/kg·BW, 400 mg/kg·BW, 800 mg/kg·BW, and T2D+water groups, respectively, n = 9).

**Figure 6:**
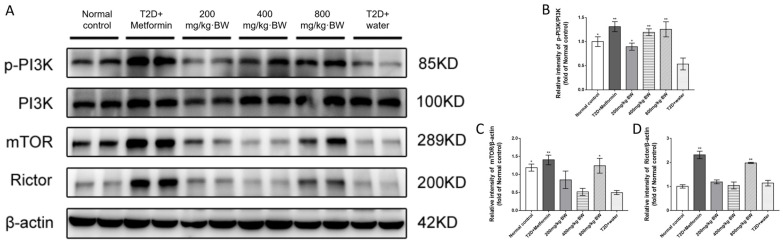
TP treatment upregulated the expression of p-PI3K, mTOR, and Rictor in each group of diabetic mice: (**A**) Western blot analysis of p-PI3K, PI3K, mTOR, Rictor, and β-actin protein levels in mouse liver; (**B**) relative expression of p-PI3K to PI3K levels; and (**C**,**D**) relative expression of mTOR and Rictor to β-actin levels, respectively. Data are expressed as means ± SD (n = 9). * *p* < 0.05 and ** *p* < 0.001 as compared to the T2D+water group.

Studies have shown that the rapamycin (TOR) kinase and its associated protein Rictor are pivotal targets for Ser473 phosphorylation in human cells. The Akt/PKB effector was suppressed by the reduction in Rictor or mammalian TOR (mTOR) expression; furthermore, the Rictor-mTOR complex could directly phosphorylate Akt/PKB on Ser473 in vitro [16]. The expression of mTOR and Rictor in diabetic mice was also examined via Western blot analysis. As shown in Figure 6, compared with the T2D+water group, the expression of mTOR and Rictor in the TP groups were significantly increased in the diabetic liver, implying that treatment with TPs might promote the expression of mTOR and Rictor to phosphorylate Akt/PKB.

**Figure 7:**
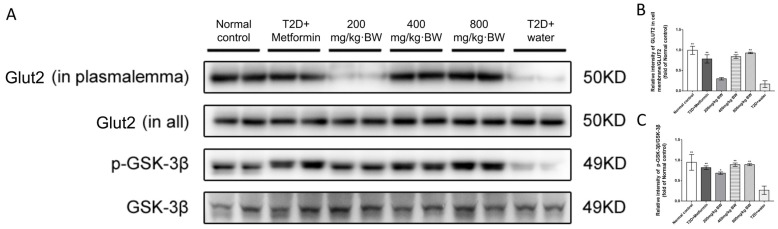
TP treatment promoted the expression of p-GSK-3β and Glut2 translocation in each group of diabetic mice: (**A**) Western blot analysis of p-GSK-3β, GSK-3β, Glut2 (in plasmalemma) and Glut2 (in all) protein levels in mouse liver; (**B**) Relative expression of p-GSK3β to GSK3β levels; (**C**) Relative expression of Glut2 (in plasma membrane) to Glut2 (in all). Data were expressed as means ± SD (n = 9). * *p* < 0.05 and ** *p* < 0.001 as compared to the T2D+water group.

The redistribution of GLUT2 promoted the uptake of glucose, which resulted in the lowering of postprandial blood glucose. Activated Akt could promote translocation, stimulated by insulin, of the glucose transporter GLUT2 to the plasma membrane of the liver cell. In our results (Figure 7A,B), after administration of TPs and metformin, the expression of GLUT2 in the plasma membrane was improved significantly compared with that in the T2D+water group. (0.78-, 0.84-, 0.9393-, and 0.16-fold of normal control mice in the T2D+metformin, 400 mg/kg·BW, 800 mg/kg·BW, and T2D+water groups, respectively, n = 9). According to our results, after administration of TPs and metformin, the expression of GLUT2 in the plasma membrane was improved significantly compared with that in the T2D+water group. (0.78-, 0.84-, 0.9393-, and 0.16-fold of normal control mice in the T2D+metformin, 400 mg/kg·BW, 800 mg/kg·BW, and T2D+water groups, respectively, n = 9).

In addition, GSK-3β, the downstream protein of Akt, was also examined. GSK-3β is a serine/threonine kinase that regulates the activity of glycogen synthase (GS). Western blot analysis showed that the protein expression of p-GSK-3β in all drug administration groups was increased significantly compared with that in the T2D+water group (0.81-, 0.68-, 0.89-, 0.89-, and 0.27-fold of normal control mice in T2D+metformin, 200 mg/kg·BW, 400 mg/kg·BW, 800 mg/kg·BW, and T2D+water groups, n = 9) in Figure 7A,C. All these results demonstrated that TPs from black tea may protect against T2D by activating the PI3K/Akt/GLUT2 signaling pathway.

## 4. Discussion and Conclusions

Numerous beneficial health effects with significant potential have been attributed to tea, the most remarkable of which is its antidiabetic activity. A study has shown that drinking tea instead of sugary beverages reduced the incidence of T2D by almost 20%, implying that tea can inhibit the onset of T2D [17]. Although polysaccharides have been proven to be the main active substance of tea to play a critical role in exhibiting antidiabetic activity, most reports have concentrated on the antidiabetic effects and underlying mechanism of green tea. There are very few studies on analyzing the hypoglycemic effect and related mechanism of polysaccharides from black tea. Research has demonstrated that the content of TPs increases with the degree of fermentation [18]; it can be speculated that TPs from black tea may possess superior antidiabetic activity.

In the present investigation, we, therefore, focused on exploring the chemical properties, antidiabetic effects, and potential mechanism of TPs from black tea, and it was demonstrated that the TP was a typical acid heteropolysaccharide with molecular weight of 67.1~70.9 kDa, which was also mainly composed of Alduronic acid with little molar content of glucose and galactose. Moreover, administration with TPs can prevent T2D from developing in HFD/STZ-induced C57BL/6J mice, and this effect was mediated by multiple factors. It was observed that after HFD/STZ induction for 60 days, treated mice exhibited thirst, polyuria, polyphagia, weight loss, hyperglycemia, insulin resistance, and dyslipidemia, all of which are the key features of T2D. Treatment with TPs had a significant adjustment effect on the levels of FBG, HOMA-IR, HOMA-IS, and blood lipids in serum. Moreover, treatment with TPs could significantly reinstate the signal pathway associated with Akt/mTOR/GLUT2 and make it nearly normal.

In addition, the results also showed that metformin could effectively inhibit blood glucose elevation and alleviate insulin resistance. Metformin is a widely used drug that can treat diabetes; it can effectively improve insulin-mediated glycogen synthesis and inhibit gluconeogenesis in liver cells [19]. Recent and more epidemiological studies have indicated that the administration of metformin could reduce the incidence, recurrence, and mortality of different types of diabetes in T2D patients.

mTOR is a highly evolutionarily conserved protein kinase that is critical for integrating cell growth factors, nutrients, and energy states. In addition, mTOR is a catalytic subunit in two distinct multiprotein complexes: mTORC1 and mTORC2 [20]. mTORC2 mainly includes mTOR kinase, a rapamycin-insensitive partner of mTOR (Rictor), a mammalian stress-activated protein kinase interacting protein 1 (mSIN1), a protein in Rictor (Protor), and mammalian lethal with SEC13 protein 8 (mLST8), as well as Dishevelled, EGL-10, and pleckstrin (DEP) domains and specific interaction with mTOR (DEPTOR). Among them, Rictor, which is a scaffold protein, can effectively promote the interaction of mTORC2 with its subunits and regulators. Furthermore, if Rictor was ablated, mTORC2 signaling would be diminished or even disrupted [21]. Akt is a downstream target of mTORC2 signaling. Rictor and mTOR were detected to fully clarify the underlying molecular mechanisms of tea polysaccharides of Akt/mTORC2 and the activation of Akt kinase. The results showed that their biological activity was positively regulated in the liver tissue of mice. The existing study suggests that metformin may similarly target the mTORC2 pathway and lead to phosphorylation of Akt [22], which is consistent with our experimental results.

Cell viability may be increased by the activation of Akt kinase with several downstream signaling pathways, including FoxOs, GSK-3β/β-catenin signaling activation, and Bax inactivation [23,24]. Among them, GSK-3β/β-catenin signaling activation is our focus because GSK-3β is one of the key proteins that directs glycogen synthesis in cells and is directly upstream of glycogen synthase (GS); it has a positive effect on regulating glucose in the blood. Our experimental results also demonstrate that tea polysaccharides can increase the activity of GSK-3β via phosphorylation of Akt. The liver is one of the most insulin-sensitive tissues; thus, in adjusting the fuel metabolism of the whole body, the mouse liver was chosen as the main experimental organ tissue. Simultaneously, Akt is a serine/threonine kinase that also works in insulin. Insulin exerts its effect mainly through the insulin receptor substrate/phosphoinositide3-kinase/Akt pathway [25]. Moreover, Akt translocates glucose transporter-4 proteins contained in intracellular vesicles to the plasma membrane through phosphorylation. This process facilitates fixing glucose in the cell [26]. In insulin resistance, there is PI3K/Akt/GLUT2 signaling in the liver and several other tissues [27]. According to known reports, metformin recovers PI3K/Akt/GLUT2 signaling and several other pathways in the liver of T2D-exposed animals [28]. Our study suggests that the hypoglycemic effects of tea polysaccharides are similar to those of metformin, and it is likely that tea polysaccharides also promote blood glucose absorption and glycogen synthesis by activating the mTOR/Akt pathway.

Phosphorylation is a key modification of signaling pathways stimulated by insulin. Many relevant important participants in insulin receptors, such as insulin receptor substrates 1 and 2, Akt, GSK-3β, and PI3K and other pathways, are either kinases themselves and/or phosphorylated via insulin stimulation [29,30]. Many of these phosphorylation effects conduct their biological activity correspondingly, mediating stepwise signaling downstream to act in concert with many functions of insulin. Particularly, a hallmark of type 2 diabetes was induced by insulin resistance and weakening of sensitivity in the liver; finally, elevated insulin no longer inhibited glucose production [31]. This is consistent with our study; after treatment with TPs or metformin, the phosphorylation levels of Akt, PI3K, and GSK-3β were significantly improved, indicating that TPs can also activate the Akt signaling pathway, similar to metformin, thereby alleviating the symptoms of diabetes in mice.

In conclusion, our present study demonstrated that the natural bioactive TP extracted and purified from black tea was a typical acid heteropolysaccharide with a molecular weight of 67.1~70.9 kDa. It was mainly composed of Alduronic acid with little molar content of glucose and galactose. Furthermore, intake of TPs exhibited significant hypoglycemic effects. The effects were, at least in part, related to the upregulation of PI3K/Akt/GSK-3β and mTOR/Rictor signaling pathways, which resulted in activating GLUT2 expression and, therefore, played a protective role in the liver. In addition, our experimental findings also illustrated that the hypoglycemic activities of TPs were similar to metformin, indicating that TPs are promising new chemical entities for further development into potent T2D therapeutics. As far as we know, this is the first study to explore the underlying mechanism of TPs from black tea in alleviating T2D through targeting PI3K/Akt/GLUT2 signaling, which could give insightful understanding of the hypoglycemic function of TPs and provide a new direction for the application of TP-related products.

## Figures and Tables

**Table 1:** Monosaccharide compositions of TPs (n = 3).

Monosaccharide Name	Content of Monosaccharide (%)	Monosaccharide Name	Content of Monosaccharide (%)
Arabinose	0.07 ± 0.03	Mannose	0.30 ± 0.03
Fucose	0.05 ± 0.01	Ribose	0.20 ± 0.02
Fructose	0.08 ± 0.01	Xylose	0.53 ± 0.07
Galactose	1.04 ± 0.28	Galacturonic acid	0.31 ± 0.03
Glucose	1.92 ± 0.74	Alduronic acid	77.80 ± 1.89

**Table 2:** Molecular parameters of three components in TPs (n = 3).

Component	Weight-Average Molecular Weight/g·mol^−1^	Number-Average Molecular Weight/g·mol^−1^	Polydispersity (aMw/bMn)	RMS Radius of Gyration/nm
TP-A	7.09 × 10^4^ (±3.33%)	1.01 × 10^4^ (±23.21%)	7.00(±23.44%)	25.50 (±48.20%)
TP-B	6.75 × 10^4^ (±1.84%)	1.10 × 10^4^ (±8.77%)	6.17 (±8.97%)	28.00 (±19.80%)
TP-C	6.71 × 10^4^ (±1.00%)	1.42 × 10^4^ (±3.00%)	4.72 (±3.16%)	27.90 (±8.20%)

aMw: weight-average molecular weight; bMn: number-average molecular weight.

## Data Availability

The original contributions presented in the study are included in the article, further inquiries can be directed to the corresponding authors.
